# Supplementary material for: Using 2D video-based pose estimation for automated prediction of autism spectrum disorders in young children
Source: Sci Rep. 2021 Jul 23;11:15069. doi: 10.1038/s41598-021-94378-z (PMC8302646; doi:10.1038/s41598-021-94378-z)
Supplement: Supplementary file 1 — Supplementary Information. [file 41598_2021_94378_MOESM1_ESM.pdf]

Supplementary Material

Participants. The Table S1 summarizes the clinical characteristics of the Training and Testing 1 samples as well as additional Testing 2 sample that was used for validation of the model. Our initial sample of TD children included 68 children (28F). Then we selected the sample of 68 children with ASD to match the TD sample with regards to age and gender. Both samples (TD and children with ASD) were divided in two so that 34 children with ASD and 34 children with TD composed Training and Testing set respectively. Children with ASD included in both samples showed a moderate to a high level of autistic symptoms, as illustrated with their average ADOS-CSS of 7.47 and 7.88, respectively. No significant differences in all used clinical measures were found between Training and Testing 1 sample (see Table S1). The Testing 2 sample is composed of the videos that were available in our cohort but who were not entered in the initial sample due to the fewer number of available videos from TD children. The children in this sample were predominantly males (1F), presenting a moderate to severe levels of symptoms of autism (mean of 7.54) and mean cognitive scores of 74.8.

**Measures**	Training set (n = 68)		Testing set 1 (n = 68)		p value	Testing set 2
	ASD (n = 34)	TD (n = 34)	ASD (n = 34)	TD (n = 34)		ASD (n = 101)
	n		n			n
Gender	14F / 20M	14F / 20M	14F / 20M	14F / 20M		1F / 100M
Modules (MT / M1 / M2)	23 / 33 / 12		24 / 31 / 13		0.95[a]	26 / 58 / 17
	Mean(SD)	*Mean(SD)*	*Mean(SD)*	*Mean(SD)*		*Mean(SD)*
Age	2.89±0.879	2.60±0.881	2.71±0.972	2.51±1.06	0.407[b]	3.46±1.16
Total Symptom Severity Score (CSS)	7.47±1.76	1.00±0.00	7.88±1.81	1.06±0.239	0.669[b]	7.54 1.86
Social Affect (SA-CSS)	6.50±1.93	1.03±0.174	7.06±2.09	1.12±0.409	0.630[b]	6.81± 2.09
Repetitive Behaviors & Restricted Interests (RRB CSS)	8.82±1.57	1.61±1.46	8.68±1.70	2.35±2.00	0.731[b]	8.62 ±1.46
Best Estimate IQ	77.3±26.5	119±18.5	70.4±27.0	115±15.1	0.529[b]	74.8± 24.1
VABS-II Adaptive Behavior	75.9±11.1	101±8.25	76.8±11.5	103±7.39	0.533[b]	77.3 ±11.4
VABS-II Communication	72.8±15.5	104±9.68	74.3±15.3	104±10.7	0.768[b]	75.4 ±15.4
VABS-II Daily Living Skills	80.4±12.6	102±8.77	79.5±13.6	103±,7.29	0.632[b]	81.4 ±12.3
VABS-II Socialization	76.1±9.02	100±8.16	78.4±10.1	102±6.62	0.309[b]	76.7± 9.99
VABS-II Motor Skills	86.6±12.8	100±8.27	86.4±12.9	102±10.5	0.668[b]	87.3± 12.8

Note. p values are obtained using Fisher's[a] exact test or nonparametric Mann-Whitney[b] tests of differences between the Training and Testing set 1.

Table S1. Description of the Training and two Testing sets of videos used in the present study.

Clinical Measures/Behavioral Phenotype. A direct measure of autistic symptoms was obtained using Autism Diagnostic Observation Schedule-Generic ADOS-G, [1] or Autism Diagnostic Observation Schedule-2nd edition (ADOS-2) [2]. Only one module is administered at one time. The latest version of ADOS allows to severity comparison scores ranging 1-10 that are aimed to be relatively independent on the participant's characteristics such as age or verbal functioning [3]. For subjects who were administered the older version of the ADOS (ADOS-G), the scores were recoded according to the revised ADOS algorithm [4] to ensure comparability with the ADOS-2. Besides comparison severity scores that indicate the overall severity of autistic symptoms [3, 5] we also included the severity scores according to domains of social affect (SA) and restricted and repetitive behaviors (RRB) thus allowing for a more precise insight on the separate dimension of autistic symptoms [6].

Cognitive functioning was assessed using various assessments depending on the age of the children. We defined the Best Estimate Intellectual Quotient following [7, 8, 9, 10, 11]. For the majority of children Psycho-Educational Profile, third edition, Verbal/Preverbal Cognition scale (PEP-3; VPC, [12]) (n = 49) was administered. We derived the VPC Developmental Quotient (DQ) by dividing the age equivalent scores by the chronological age of the child. For a smaller subset of children (younger than 2 years old) when PEP-3 was not administered we used Mullen Early Learning scales [13], (n = 14). Here again, developmental quotients were obtained using the mean age equivalent scores from four cognitive scales (Visual Reception, Fine Motor, Receptive Language and Expressive Language) and divided by chronological age.

Adaptive functioning was assessed using the Vineland Adaptive Behavior Scales, second edition (VABS-II; [14]). This standardized parents interview measures adaptive functioning from childhood to adulthood in the domains of communication, daily-living skills, socialization, and motor domain. In each domain, standardized scores (SStd) are denoted by adaptive level,

ranging from low to high. The four domain standardized scores are then combined to yield the adaptive behavior composite score as a global measure of adaptive functioning of an individual.

Neural Network Training. The neural network hyper-parameter tuning was done by taking a small sub-sample of 50 OpenPose processed videos videos (25 ASD and 25 TD). We tested a ResNet50 CNN ([15]) for as the first CNN architechture for extraction of high dimensional features and a 512 LSTM unit recurrent neural network([16]) to add the temporal dimension for making video classification neural network. Out first test involved splitting the videos into segments of length 5, 10 and 15 seconds and monitored the training and validation loss to check for overfitting. We observed 5 second segments to provide us with the least validation loss among the 3 tests (see Fig.S2A). We further tested for improvements in the validation loss when the video dataset was downsampled to the resolution 320X240 from 696X554 resolution (see Fig.S2B) and observed a better fit for downsampled 320X240 videos.

After conducting the previous tests we observed that a batch size of 256 or 128 was optimal to achieve the least validation loss. We also tested out several convolutional neural network architectures for feature extraction, excluding fully connected dense layers (see Fig.S2, panels C-D), out of which VGG16 ([17]) gave substantially better results compared to other CNN feature extraction methods such as ResNet50 ([15]). We also tried to use a bidirectional LSTM ([18]) in order to check for any further decrease in validation loss. However, using a bidirectional LSTM led to over-fitting in the neural network (see Fig.S2E).

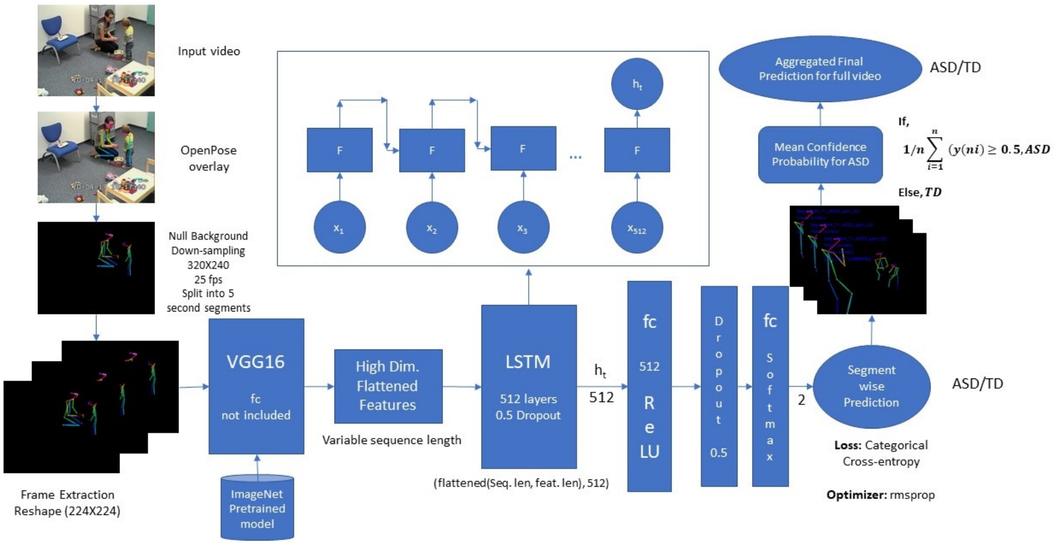

Figure S1. VGG16 LSTM detailed architecture representing pre-processing, feature extraction, addition of temporal dimension to features, segment-wise classification and aggregation of prediction over the entire video set to give final prediction.

Thus we observed the best model for our dataset to be a VGG16 LSTM at 128 batch size, 100 epochs and 320X240 resolution video with a validation loss of 0.301. We carry out the training with this configuration at 80-20 and 70-30 splits and observed a better performance with 80-20 split. Fig.S3. A detailed architecture of the VGG16 LSTM neural network can also be represented by Fig.S1.

Predicted class attribution across filming settings. The ADOS videos in the context of our longitudinal cohort were acquired using different systems. The majority of the videos was acquired using remotely controlled dome camera system. This setting involves cameras fixed at the height of 2 meters from the floor, and allowed manual pan, tilt, zoom as well as flexible switch between two camera views. This camera system was used in 44 videos from the training sample and 48 videos of the Testing set 1. The other settings included a fixed angle gopro camera that was positioned either high (above 160 cm of the floor) (Training: 10 videos, Testing:9 videos) either low (70-80cm from the floor) (Training: 14, Testing 11 videos). The accuracy of prediction across settings was the highest in a low positioned fixed angle gopro cameras, followed by the accuracy value of 81% in the setting involving high positioned manually controlled cameras. Finally, the poorest accuracy (77%) characterized the setting with a fixed cameras and high filming angle (see Fig.S4).

In addition to testing the relation with general severity of symptoms of autism we also wanted to obtain the appreciation of the relation of ASD probability with the 27 individual symptoms that are coded across the three used ADOS modules (the list of symptoms included in this analysis is shown in Table S2). Of note, individual symptoms of autism in ADOS are scored on a 4

point scale ranging from 0 ("no evidence of abnormality") to 3 ("markedly abnormal"). With the exception of two symptoms (Name calling and Response to joint attention) that are coded during a specific social press the other symptoms codes are based on the overall behavioral sample obtained during the ADOS session. Based on clinical experience and knowledge and coding guidelines of the ADOS the clinician attributes a code from 0 to 3 to code the presence of abnormality. For example, to obtain a code of 0 (no abnormality) for the item Unusual Eye Contact the child must show the use of the eye contact that is "clear, flexible, socially modulated and appropriate" and this judgment is only possible after the child is given an important amount of occasions to express his/her requests, responds to request of others, share his/her interest for the proposed activities. Similarly, a child who receives a code of 0 on the item Showing is the child who was able to show toys/objects by "holding them up or placing them in front of others and using the eye contact with or without the vocalization" in a minimum two activities.

BEHAVIORS	Toddler Module	Module 1	Module 2
**A Communication**			
Frequency of Spontaneous Vocalization Directed to Others	☑	☑	☑*
Intonation of Vocalizations or Verbalizations	☑	☑	☑
Immediate Echolalia	☑	☑	☑
Stereotyped/Idiosyncratic Use of Words or Phrases	☑	☑	☑
Use of Another's Body	☑	☑	☐
Pointing	☑	☑	☑
Gestures	☑	☑	☑
**B Reciprocal Social Interaction**			
Unusual Eye Contact	☑	☑	☑
Facial Expressions Directed to Others	☑	☑	☑
Integration of Gaze and Other Behaviors during Social Overtures	☑	☑	☐
Shared Enjoyment in Interaction	☑	☑	☑
Response to Name	☑	☑	☑
Requesting	☑	☑	☐
Showing	☑	☑	☑
Giving	☑	☑	☐
Spontaneous Initiation of Joint Attention	☑	☑	☑
Response to Joint Attention	☑	☑	☑
Quality of Social Overtures	☑	☑	☑
Amount of Social Overtures/Maintenance of Attention: Examiner	☑	☑*	☑*
Quality of Social Response	☑	☑*	☑
Overall Quality of Rapport	☑	☑*	☑
**C Play**			
Functional Play with Objects	☑	☑	☑
Imagination / Creativity	☑	☑	☑
**D Stereotyped Behaviors and Restricted Interests**			
Unusual Sensory Interest in Play Material/Person	☑	☑	☑
Hand and Finger and Other Complex Mannerisms	☑	☑	☑
Self-Injurious Behavior	☑	☑	☑
Unusually Repetitive Interests or Stereotyped Behaviors	☑	☑	☑

* Item not included in the given module of the older version (ADOS-G) of the schedule.

Table S2. Selected items from the gold-standard diagnostic assessment, the ADOS-G [1] and ADOS-2 [2]. The different columns correspond to different modules the choice of which is performed as a function of age and language level.

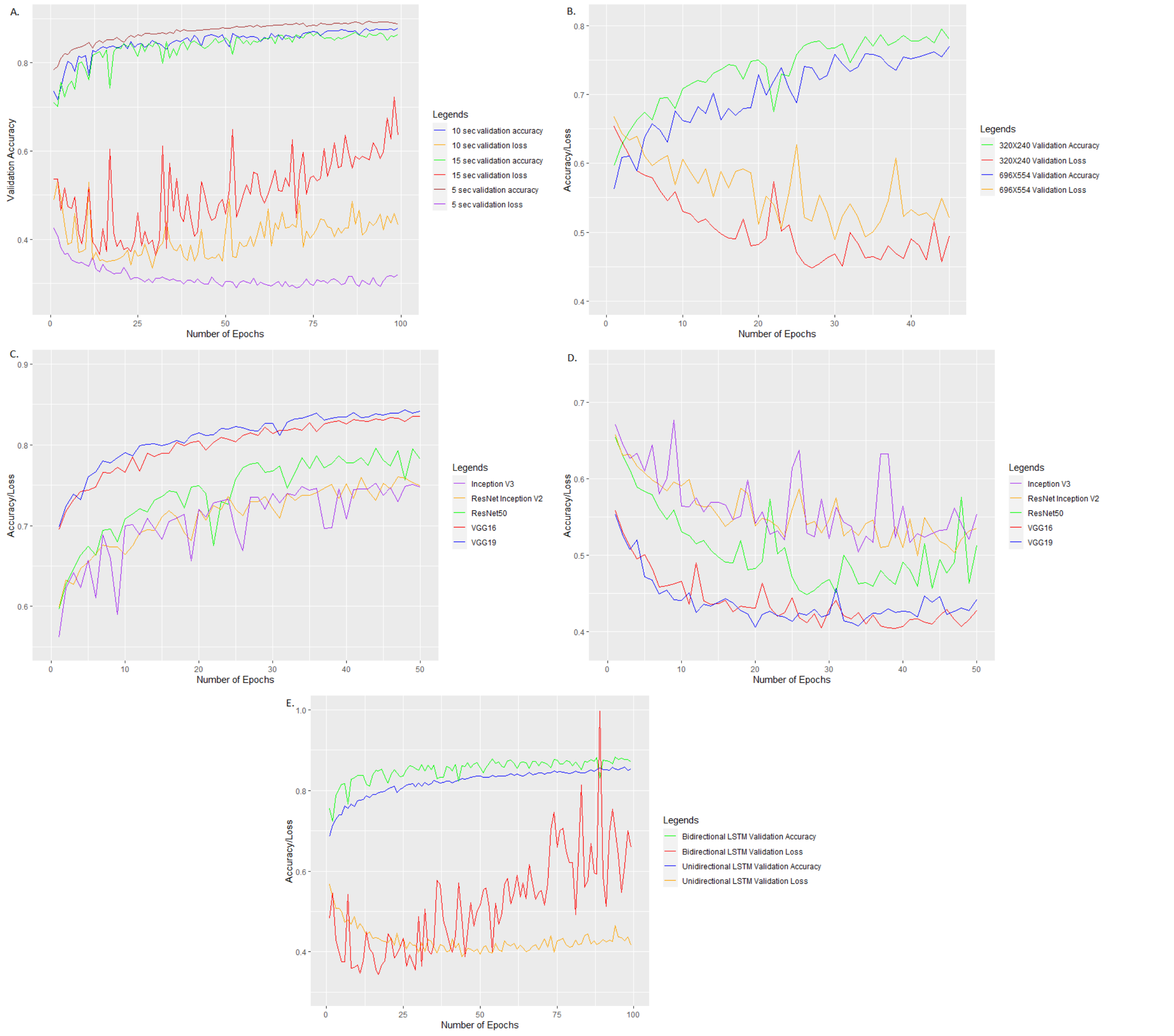

Figure S2. A. 5 sec, 10 sec and 15 second training and validation plots for ResNet50 LSTM at 256 batch size, 100 epochs and 70-30 training-validation split, B. 320X240 vs 696X554 resolution videos training and validation plots for ResNet50 LSTM, 256 batch size, 50 epochs, 70-30 training-validation split, C. Training and validation accuracy for different High Dimensional feature extraction CNN models with LSTM RNN model at 256 batch size, 50 epochs and 70-30 training-validation split, D. Training and validation accuracy for different High Dimensional feature extraction CNN models with LSTM RNN model at 256 batch size, 50 epochs and 70-30 training-validation split, E. Training and validation accuracy and loss for ResNet50 LSTM and ResNet50 Bidirectional LSTM at 100 epochs,256 batch size and 70-30 training-validation split

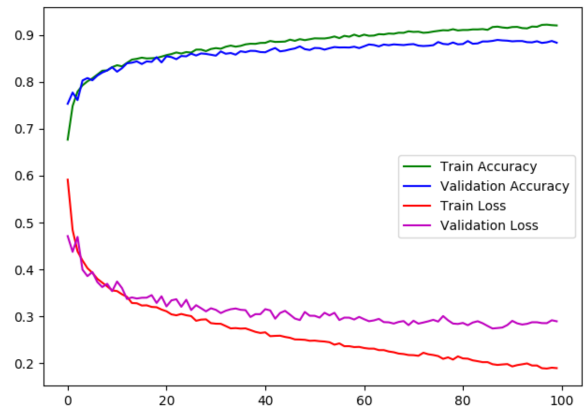

Figure S3. Training Accuracy, Training Loss, Validation Accuracy and Validation loss with 68 balanced training video dataset at 80-20 split, 128 Batch Size, 320X240 resolution and 100 Epochs for VGG16 LSTM neural network.

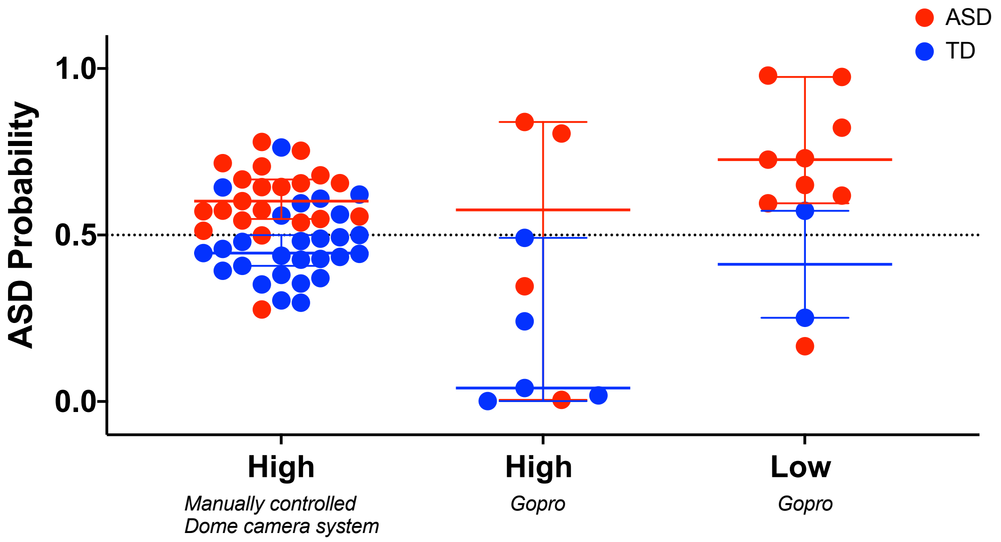

Figure S4. Scatter plots depicting ASD probability across the three setting types (columns) and for the two diagnostic groups (typically developing in blue and children with ASD in red). In high positioned manually controlled dome camera system the accuracy was 81%; in the setting involving gopros and high filming angle the accuracy was 77%; Finally, in the setting involving gopros positioned at low height filming angle the accuracy was 82%.

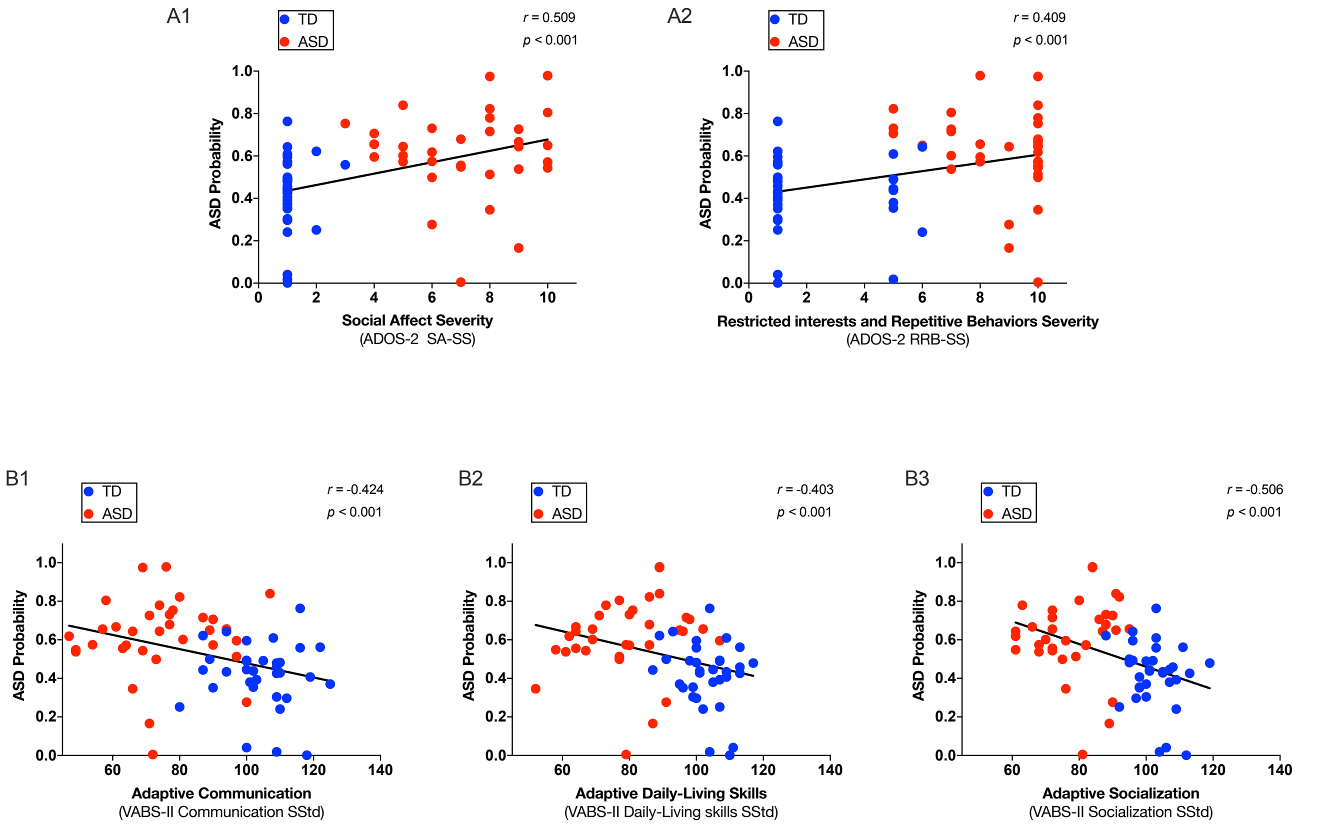

Figure S5. Relation between predicted ASD probability and subdomains of (A) Autism Diagnosis Observation Schedule-ADOS; (B) Vineland Adaptive Behavior Scales (p values were considered significant if $p < 0.008$ after applying Bonferroni corrections for multiple comparisons). A1. Social Affect Severity Score, A2. Restricted interests and Repetitive Behaviors-RRB Severity Score; B1. Communication domain; B2. Socialization domain;B3. Daily Living Skills domain. The least squares linear fit is depicted as black line and values of Spearman r coefficient and corresponding p values are shown on each graph.

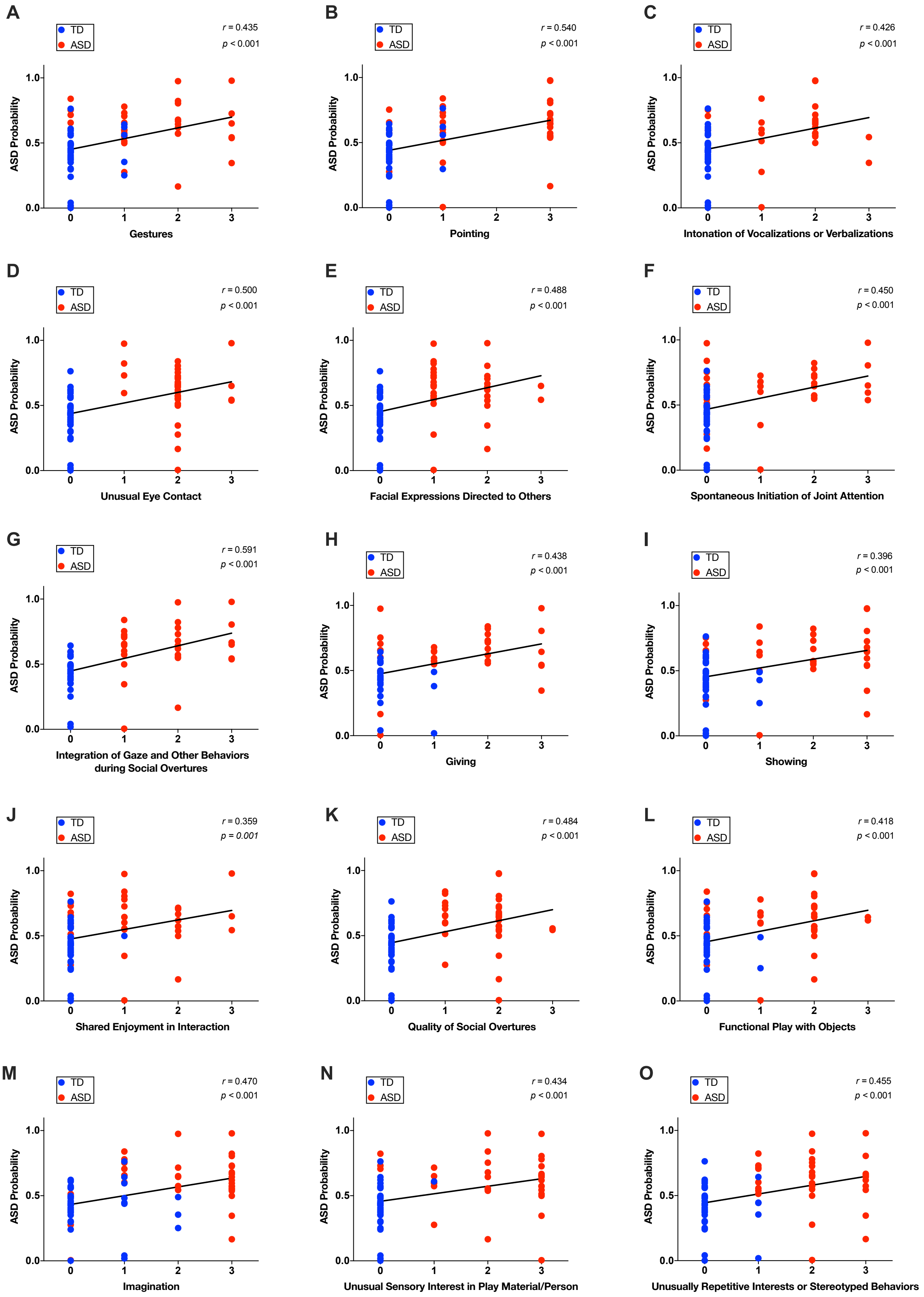

Figure S6. Relation between predicted ASD probability and individual symptoms derived from ADOS ($p < 0.002$ after applying Bonferroni corrections for multiple comparisons). A-C Communication domain; D-K Reciprocal Social Interaction domain; L-M Play; N-O Repetitive and Restricted behaviors domain of ADOS. The least squares linear fit is depicted as black line and values of Spearman r coefficient and corresponding p values are shown on each graph.

References

1. Lord, C. *et al.* The autism diagnostic observation schedule-generic: a standard measure of social and communication deficits associated with the spectrum of autism. *J. Autism Dev. Disord.* **30**, 205–223 (2000).
2. Lord, C. *et al.* *Autism diagnostic observation schedule: ADOS-2* (Western Psychological Services, Los Angeles, Calif., 2012). OCLC: 851410387.
3. Gotham, K., Pickles, A. & Lord, C. Standardizing ADOS scores for a measure of severity in autism spectrum disorders. *J. Autism Dev. Disord.* **39**, 693–705, DOI: 10.1007/s10803-008-0674-3 (2009). ArXiv: NIHMS150003 ISBN: 0162-3257.
4. Gotham, K., Risi, S., Pickles, A. & Lord, C. The autism diagnostic observation schedule: Revised algorithms for improved diagnostic validity. *J. Autism Dev. Disord.* **37**, 613–627, DOI: 10.1007/s10803-006-0280-1 (2007). ISBN: 0162-3257.
5. Estes, A. *et al.* Behavioral, cognitive, and adaptive development in infants with autism spectrum disorder in the first 2 years of life. *J. Neurodev. Disord.* **7**, 24, DOI: 10.1186/s11689-015-9117-6 (2015).
6. Hus, V., Gotham, K. & Lord, C. Standardizing ADOS domain scores: Separating severity of social affect and restricted and repetitive behaviors. *J. Autism Dev. Disord.* **44**, 2400–2412, DOI: 10.1007/s10803-012-1719-1 (2014). ISBN: 0162-3257.
7. Howlin, P., Savage, S., Moss, P., Tempier, A. & Rutter, M. Cognitive and language skills in adults with autism: a 40-year follow-up. *J. Child Psychol. Psychiatry* **55**, 49–58, DOI: 10.1111/jcpp.12115 (2014).
8. Kojovic, N., Ben Hadid, L., Franchini, M. & Schaer, M. Sensory Processing Issues and Their Association with Social Difficulties in Children with Autism Spectrum Disorders. *J. Clin. Medicine* **8**, 1508, DOI: 10.3390/jcm8101508 (2019).
9. Howlin, P., Moss, P., Savage, S. & Rutter, M. Social Outcomes in Mid- to Later Adulthood Among Individuals Diagnosed With Autism and Average Nonverbal IQ as Children. *J. Am. Acad. Child & Adolesc. Psychiatry* **52**, 572–581.e1, DOI: 10.1016/j.jaac.2013.02.017 (2013). Publisher: Elsevier.
10. Bishop, S. L., Farmer, C. & Thurm, A. Measurement of Nonverbal IQ in Autism Spectrum Disorder: Scores in Young Adulthood Compared to Early Childhood. *J. Autism Dev. Disord.* **45**, 966–974, DOI: 10.1007/s10803-014-2250-3 (2015).
11. Liu, X.-Q., Paterson, A. D. & Szatmari, P. Genome-wide Linkage Analyses of Quantitative and Categorical Autism Subphenotypes. *Biol. Psychiatry* **64**, 561–570, DOI: 10.1016/j.biopsych.2008.05.023 (2008).
12. Schopler, E. *PEP-3: Psychoeducational Profile* (PRO-ED, 2005).
13. Mullen, E. M. *Mullen Scales of Early Learning Manual* (American Guidance Service, Circle Pines, Minnesota, 1995).
14. Sparrow, S. S., Balla, D. & Cicchetti, D. V. *Vineland II: Vineland Adaptative Behavior Scales : Survey Forms Manual : a Revision of Hte Vineland Social Maturity Scale by Edgar A. Doll* (Pearson, 2005).
15. He, K., Zhang, X., Ren, S. & Sun, J. Deep Residual Learning for Image Recognition. *arXiv:1512.03385 [cs]* (2015). ArXiv: 1512.03385.
16. Hochreiter, S. & Schmidhuber, J. Long Short-Term Memory. *Neural Comput.* **9**, 1735–1780, DOI: 10.1162/neco.1997.9.8.1735 (1997).
17. Simonyan, K. & Zisserman, A. Very Deep Convolutional Networks for Large-Scale Image Recognition. *arXiv:1409.1556 [cs]* (2015). ArXiv: 1409.1556.
18. Zeyer, A., Doetsch, P., Voigtlaender, P., Schlüter, R. & Ney, H. A Comprehensive Study of Deep Bidirectional LSTM RNNs for Acoustic Modeling in Speech Recognition. *2017 IEEE Int. Conf. on Acoust. Speech Signal Process. (ICASSP)* 2462–2466, DOI: 10.1109/ICASSP.2017.7952599 (2017). ArXiv: 1606.06871.
